# Proteome changes during *in vitro* culture adaptation of *Toxoplasma gondii* archetypal II and III field isolates

**DOI:** 10.3389/fcimb.2025.1633384

**Published:** 2025-09-16

**Authors:** Joachim Müller, Javier Regidor-Cerrillo, David Arranz-Solís, Sophie Braga-Lagache, Anne-Christine Uldry, Manfred Heller, Rafael Calero-Bernal, Andrew Hemphill, Luis Miguel Ortega-Mora

**Affiliations:** ^1^ Institute of Parasitology, Vetsuisse Faculty, University of Bern, Bern, Switzerland; ^2^ SALUVET-INNOVA, Animal Health Department, Faculty of Veterinary Sciences, Complutense University of Madrid, Madrid, Spain; ^3^ SALUVET, Animal Health Department, Faculty of Veterinary Sciences, Complutense University of Madrid, Madrid, Spain; ^4^ Proteomics and Mass Spectrometry Core Facility, Department for BioMedical Research (DBMR), University of Bern, Bern, Switzerland

**Keywords:** *Toxoplasma gondii*, proteome, culture adaptation, bradyzoite, virulence factors, drug- vaccine targets

## Abstract

**Introduction:**

Rapid *in vitro* culture adaptation of recently obtained *Toxoplasma gondii* isolates leading to deep changes in relevant phenotypic traits has been demonstrated earlier. Few reports exist on the molecular bases that govern this adaptation. Herein, we analyzed the *T. gondii* proteomes of different isolates at two timepoints during cell culture adaptation.

**Methods:**

The differential proteomes of six recently obtained archetypal European *T. gondii* Type II (TgShSp1 (Genotype ToxoDB#3), TgShSp2 (#1), TgShSp3 (#3) and TgShSp16 (#3)) and Type III (TgShSp24 (#2) and TgPigSp1(#2)) isolates maintained at low (10-16) and high (50-53) passage numbers in Vero cells were determined by label free liquid chromatography–mass spectrometry.

**Results:**

Among these isolates, 2.3% and 10.2% of proteins were differentially or constantly abundant when comparing low and high passage numbers. Constant proteins included components involved in essential cellular processes such as energy metabolism or protein synthesis, many of them identified as drug and vaccine targets. Interestingly, differentially abundant proteins were clearly linked to phenotypic changes associated to *in vitro* adaptation: loss of ability to spontaneously form cysts at high passages and decreased expression of cyst and bradyzoite markers (BAG1, Enolase 1, and SRS35A), while culture adaptation was associated with increased abundance of recognized virulence factors such as GRA15, GRA16, TEEGR and NSM.

**Conclusion:**

Our results highlight the changes at the proteomic level that take place in recently obtained isolates after *in vitro* culture adaptation, an important feature that should be considered during *T. gondii* investigations.

## 1 Introduction

The apicomplexan *Toxoplasma gondii* is an opportunistic intracellular protist parasite with high prevalence in animal and human populations worldwide (Wilson et al., 2024). In most cases, the infection remains asymptomatic. However, *T. gondii* may cause cerebral toxoplasmosis resulting in fatal encephalitis in immunocompromised individuals such as AIDS patients (Dian et al., 2022), reproductive failure (e.g. abortions) in pregnant women and small ruminants, and infections are frequently linked to ocular disease (Goh et al., 2022; Maenz et al., 2014; Schlüter et al., 2014). Felids, acting as specific definitive hosts, excrete oocysts in their feces; after sporulation, sporozoites are formed within oocysts rendering them orally infective. After ingestion by a wide range of species constituting intermediate hosts, excysted sporozoites invade intestinal epithelial cells and differentiate into tachyzoites. They subsequently infect/invade cells of the reticulo-endothelial system such as lymphocytes, dendritic cells and macrophages, and employ these cells for dissemination throughout the organism employing a Trojan-horse strategy. Ultimately, tachyzoites reach muscular tissue and/or the central nervous system of the host where, after the onset of immunity, they differentiate into bradyzoites, encapsulated within mature tissue cysts (Attias et al., 2020). The ingestion of tissues containing bradyzoites by a felid concludes the life cycle. Humans become infected after ingestion of food or water contaminated with sporulated oocysts shed along cat feces, undercooked meat containing tissue cysts (Madireddy et al., 2022) or – as fetuses – via transplacental transmission of tachyzoites upon a primary infection during pregnancy (Kota and Shabbir, 2023).

The majority of strains found in Europe and North America are classified into three clonal genetic lineages labelled Type I, II and III (Howe and Sibley, 1995), with genotypes from clade D prevailing in Europe (Fernández-Escobar et al., 2022). Tachyzoites of Type II and III laboratory strains, but not Type I tachyzoites, can undergo bradyzoite differentiation *in vitro* when they are grown under stress conditions (Lindsay and Dubey, 2011; Dardé, 2008). Also, the three Types traditionally differ in their growth rate, virulence, ability to cross epithelial barriers (transmigration), and capacity to form cysts (Lindsay and Dubey, 2011). Type I strains generally replicate faster and, consequently, are more virulent in mice than Type II and III strains (Howe and Sibley, 1995; Lindsay and Dubey, 2011). Nevertheless, the three Types also differ with respect to evasion-related mechanisms against host immunity (Sanchez and Besteiro, 2021).

Phenotypic and biological characterization of different *T. gondii* genetic variants has been broadly developed using reference laboratory isolates, such as RH, ME49, Prugniaud (PRU) or VEG, maintained *in vitro* for an uncontrolled number of passages. A recent study demonstrated the rapid adaptation to cell culture of recently obtained Type II and III isolates and its association with changes in parasite proliferation and loss of the capacity to spontaneously form cysts in Vero cells, together with changes - exacerbation or attenuation - in virulence in outbred mice (Colos-Arango et al., 2023). To address the question to what extent *in vitro* culture could lead to changes in the *T. gondii* proteome, we investigated six recently obtained canonical isolates of European origin, four Type II (genotypes ToxoDB#1 and #3), and two Type III (ToxoDB#2) isolates. We compared the proteomes of each of these isolates after adaptation in short-term culture *in vitro* (from passage numbers 10–16 to 50-53), focusing on differentially abundant (DA) proteins, but also on constantly abundant (CA) proteins to reveal potentially essential proteins.

## 2 Materials and methods

**Figure 1:**
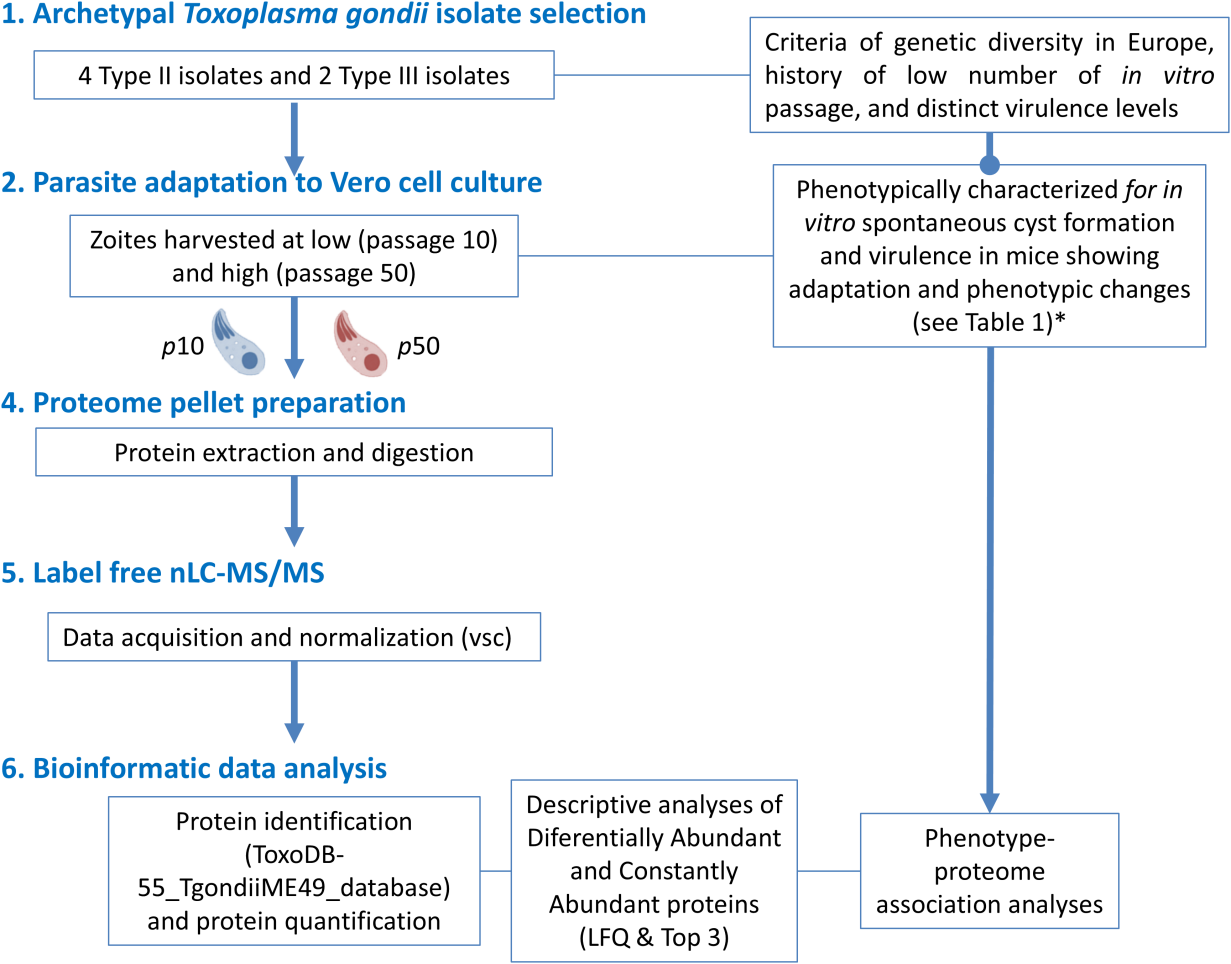
Workflow of the experimental design for proteome analyses related to the cell culture adaptation phenomena of 6 canonical *T. gondii* isolates. (*) Procedures and data obtained in Colos-Arango et al. (2023).

The experimental design layout for this work is shown in **Figure 1**.

### 2.1 *T. gondii* isolates, culture conditions, and sample production for proteomics

The *T. gondii* isolates used in this study were recently obtained from three cases of sheep abortion (TgShSp1, TgShSp2 and TgShSp3; all Type II), two chronically infected adult sheep (TgShSp16 -Type II- and TgShSp24 -Type III-), and one fattening pig at grow-finishing phase (TgPigSp1; Type III) (Fernández-Escobar et al., 2020a, 2020b, 2021). These isolates were maintained *in vitro* after one or two passages in mice during the isolation step, from where the passage number in cell culture has been strictly controlled and recorded. The isolates, genotype and phenotypic traits at low and high passage numbers were determined in a recent publication (Colos-Arango et al., 2023) and summarized in **Table 1**.

**Table 1:** *Toxoplasma gondii* isolates, genotype and phenotypic variations after 40 passages in cell culture (gathered from Colos-Arango et al., 2023).

Isolate ID^a^	Type	RFLP genotype # (ToxoDB)	Phenotypic traits *in vitro*						Phenotypic traits *in vivo*							
			Spontaneous cyst formation^b^		Tachyzoite production^c^		Tachyzoite-bradyzoite conversion rate (%)^d^		Mouse morbidity (%, clinical scoring)^e^		Mouse mortality (%)^f^		Brain median parasite load (zoites/mg tissue)^g^		Lung median parasite load (zoites/mg tissue)^h^	
			*p10*	*p50*	*p10*	*p50*	*p10*	*p50*	*p10*	*p50*	*p10*	*p50*	*p10*	*p50*	*p10*	*p50*
TgShSp1	II	#3^‡^	21.5	0*	0.6	0.6	73	0	100 (1.0)	100 (1.0)	0	0	430	7.5*	0	0
TgShSp2	II	#1	0.5	0.2	0.8	1.1*	66	0.6	0 (0.0)	100* (2.1)	0	20	25	15	1.4	0
TgShSp3	II	#3^‡^	31.8	1.3*	0.6	1.4*	69	4.2	0 (0.0)	100* (2.1)	0	30	66	335*	21	0.4
TgShSp16	II	#3^‡^	8.5	0*	0.7	1.3*	27	0	100 (3.1)	100 (2.0)	50	0*	155	11*	3.5	0*
TgShSp24	III	#2	5.8	0*	0.8	1.5*	45	0	90 (2.1)	100 (4.4)	30	100*	40	4.3*	14.1	1494*
TgPigSp1	III	#2	2.6	0.8	0.7	1.3*	66	2	100 (4.4)	100 (4.4)	100	100	322	229	12863	2248*

a ‘‘Sh” and ‘‘Pig” in the name denotes host origin, sheep (*Ovis aries*) and domestic pig (*Sus scrofa*), respectively. ‘‘Sp” in the name denotes the geographical origin of Spain.

b Mean (x 10^4^) spontaneous mature cyst numbers at low passages 10-15 (p10) and high passages 45-50 (p50) in Vero cultures inoculated with a multiplicity of infection 4:1 after egression of parasites.

c Mean (x 10^8^) zoite numbers at low passages 10-15 (p10) (at 7 and 5 days post-inoculation for Type II and Type III isolates, respectively) and high passages 45-50 (p50) (at 3.5–4 days post-inoculation) in Vero cultures inoculated with a multiplicity of infection 4:1 after egression of parasites.

d Percentage of tachyzoite-bradyzoite conversion determined by immunofluorescence assay of TgBAG1-positive vacuoles at low passages 10-15 (p10) and high passages 45-50 (p50) at 3-3.5 days post-inoculation for Type II and Type III isolates in Vero cultures inoculated with a multiplicity of infection 1:1 after egression of parasites.

e Morbidity in mice inoculated with lethal dose 50 (LD_50_); percentage of clinically affected mice, median clinical score at 14 and 42 days post-infection. Clinical scoring: asymptomatic (0), ruffle coat/ascites (1), rounded back/ascites/loss of body weight (2), loss of body condition/body weight (>10%; < 20%)/moderate ascites (3), severe loss of body weight (>20%)/gross ascites with marked abdominal distension/severe respiratory distress (continuous fast breathing and chest retraction)/neurological signs (or humane euthanasia) and sudden death (4).

f Mortality of mice inoculated with lethal dose 50 (LD_50_); percentage of death mice.

g,h Parasite burdens (zoites/mg of tissue) quantified in the brain (g) and lung (h) by 529RE quantitative PCR.

^‡^Genotype ToxoDB#3 is also known as the Type II-PRU variant.

*Denotes significant differences between p10 *vs*. p50 for each isolate.

*Toxoplasma gondii* isolates were maintained by serial passages in Vero cells (ATCC^®^ CCL81™) as previously described using Dulbecco Modified Eagle Medium (DMEM) with 1% fetal bovine serum (FBS) (Colos-Arango et al., 2023). To obtain parasite pellets for proteome analyses, each of the isolates was grown in 75 cm^2^ culture flasks with confluent Vero cells for low passage (10-16) at multiplicity of infection (MOI) ranging from 0.25:1 to 10:1, and maintained for 2 to 10 days post-infection until parasite egression (5-25%) was evident. Under the same growth conditions, parasite pellets for high passage number (50-53) were obtained from cultures at MOI 1:1 to 18:1, after 2 to 7 days post-inoculation when lysis of cell monolayer reached 5-80%. Parasites were harvested by scraping the Vero cell monolayer into 50-ml conical tubes followed by centrifugation at 700 × *g* for 7 minutes. Next, pellets were resuspended in 5 ml PBS and passed five times each through 27 and 30G needles to disrupt host cells and release zoites. To remove cell debris while diminishing the exclusion of spontaneously formed cysts, released parasites were purified by elution through a 10-μm polycarbonate filter (Isopore™, TCTP04700, Merck Millipore). The final number of purified zoites was determined by trypan blue exclusion in a Neubauer chamber, and the equivalent to 2×10^7^ parasites was added to 1.5 ml tubes to obtain the final pellets. Additionally, structures compatible with tissue cyst were also counted in Neubauer chamber in selected samples for each isolate. After centrifugation at 5000 × *g* for 5 min, the supernatant was discarded, and pellets stored at -80°C until use for protein extraction (see below) and additional cyst staining with FITC-conjugated *Dolichos biflorus* lectin (DBL-FITC, Vector Laboratories) at 1:500 dilution. Three low (10–16 passages) and high (50–53 passages) passage replicates from each isolate were used for proteomic analyses. Details on sample collection are summarized in **Supplementary Table S1**.

### 2.2 Proteomics

Proteins from pellets were extracted, reduced and alkylated as described earlier (Hänggeli et al., 2023) followed by proteome digestion as reported elsewhere (Ajiboye et al., 2024). Aliquots of 5 μL of each digest, corresponding to 500 ng, were analyzed twice with a 90-min gradient by a nano-liquid chromatography mass spectrometry system consisting of an Orbitrap Fusion LUMOS mass spectrometer that was coupled with a Dionex Ultimate 3000 nano-UPLC system (ThermoFischer Scientific, Reinach, Switzerland). A standard data-dependent acquisition method as described elsewhere (Buchs et al., 2018) was used with a homemade AcquityTM CSH C18 (Waters, Baden, Switzerland) separation column (1.7 μm, 130 Å, 75 μm × 20 cm) at a flow rate of 250 nL/min.

Mass spectrometry data were processed by MaxQuant software version 1.6.14.0 against the ToxoDB-55_TgondiiME49_AnnotatedProteins database, to which common contaminants were added. Match between runs was enabled with a matching time window of 0.7 min but prevented across cell lines by using non-consecutive fraction numbers. A strict trypsin cleavage rule was applied, allowing for up to three missed cleavages, variable modifications of protein N-terminal acetylation and oxidation of methionine, and static modification of cysteine with carbamidomethylation. Precursor and fragment mass tolerances were set to 10 ppm and 0.4 Da respectively. Peptide spectrum matches, as well as peptide and protein group identifications, were filtered to a 1% false discovery rate (FDR) based on reversed database sequence matches, and a minimum of two razor or unique peptides were required to accept a protein group identification. The comparison of protein abundance between groups was made using both MaxQuant’s Label-Free Quantification (LFQ) values as well as Top3 values (sum of the 3 most intense peptide form intensities), as reported elsewhere (Hänggeli et al., 2023). Protein identifications from the contaminants database (e.g., trypsin or BSA) as well as proteins identified only by site were removed for statistical validation.

### 2.3 Statistics

Proteome data were analyzed using two different approaches, namely analysis of differential abundance and of equivalence at low *vs.* high passage numbers for each strain. For each strain and passage level, three biological replicates were analyzed. Peptides from all replicates were first normalized by the Variance Stabilizing Normalization (vsn) method (Välikangas et al., 2018). Based on imputed Top3 (iTop3) and LFQ (iLFQ) values, differential abundance tests were performed by applying the Empirical Bayes test on protein-iTop3 and iLFQ. For each parameter, significance was defined as a minimal log2 fold change (LFC) of 1 and a maximum adjusted p-value (FDR-controlled Benjamini and Hochberg multiple test correction) of 0.05. To strengthen our analysis, only proteins with significantly different levels between low and high passages in both iTop3 and iLFQ values were regarded as significant DA proteins and referred to as “up- or downregulated DA proteins” in the script (*i.e.* high passage number *vs*. low passage number). The equivalent tests between low and high passages were performed on the complete data, i.e., only if a given protein was seen in all replicates of each group, and not on imputed data, by two one-sided t-tests: one testing for LFC > -1, and one testing for LFC < 1; the largest p-value of each test is reported, and corrected for multiple testing. Additionally, equivalence was defined as significant based on a LFC of 1 and a maximum adjusted p-value of 0.05. As for the DA proteins, only proteins with equivalence in both LFQ and Top3 were regarded as significantly equivalent proteins and referred to as CA proteins in the script. Proteins not falling into either of these categories are referred to as “variable”.

A Pearson correlation analysis was also performed to evaluate the similarities between proteomic profiles across experimental groups. For each group, the mean log2-transformed protein abundance was calculated across all biological replicates. Pairwise Pearson correlation coefficients (r values) between groups were computed in R (v 4.4.0) using the cor() function with method = “pearson”. The resulting correlation matrix was visualized as a heatmap using the ComplexHeatmap package (v. 2.21.1) (Gu et al., 2016). Clustering analysis was also performed to visualize the closer proteomes among *T. gondii* isolates at high and low passages.

## 3 Results

### 3.1 Overall proteome analysis of *Toxoplasma gondii* canonical isolates demonstrates limited variation on proteome abundances

**Figure 2:**
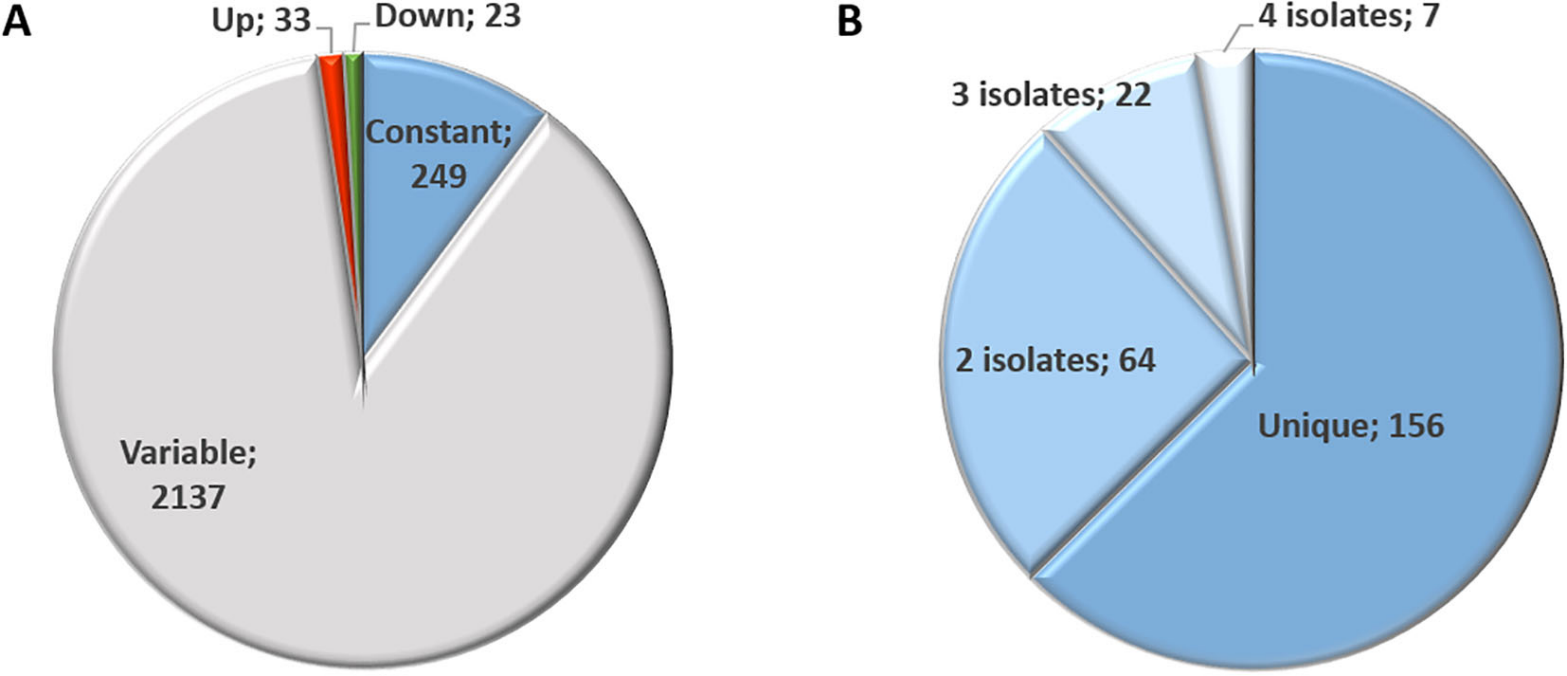
Proteome overview of *T. gondii* Type II and Type III canonical isolates **(A)** and overview of identified constantly abundant (CA) proteins **(B)**. Number indicates the number of proteins identified for each category.

Overall, proteome analysis of the isolates TgShSp1, TgShSp2, TgShSp3, TgShSp16, TgShSp24 and TgPigSp1 yielded 23,361 unique peptides matching to 2,442 proteins. The complete dataset including differentially abundant (DA) and equivalent proteins is compiled in **Supplementary Table S2**. Only 56 of these 2,442 proteins (2.3%) were significantly DA at high compared to low passages among *T. gondii* isolates, of which 33 presented higher and 23 lower abundance levels. A total of 249 proteins (10.2%) were regarded as constantly abundant (CA), while the remaining 2,137 proteins (87.5%) were considered as “variable”, meaning that abundance was fluctuating but not significantly different between high and low passage numbers (**Figure 2A**).

### 3.2 Constantly abundant proteins detected in Type II and III canonical isolates encompass relevant targets for intervention

**Figure 3:**
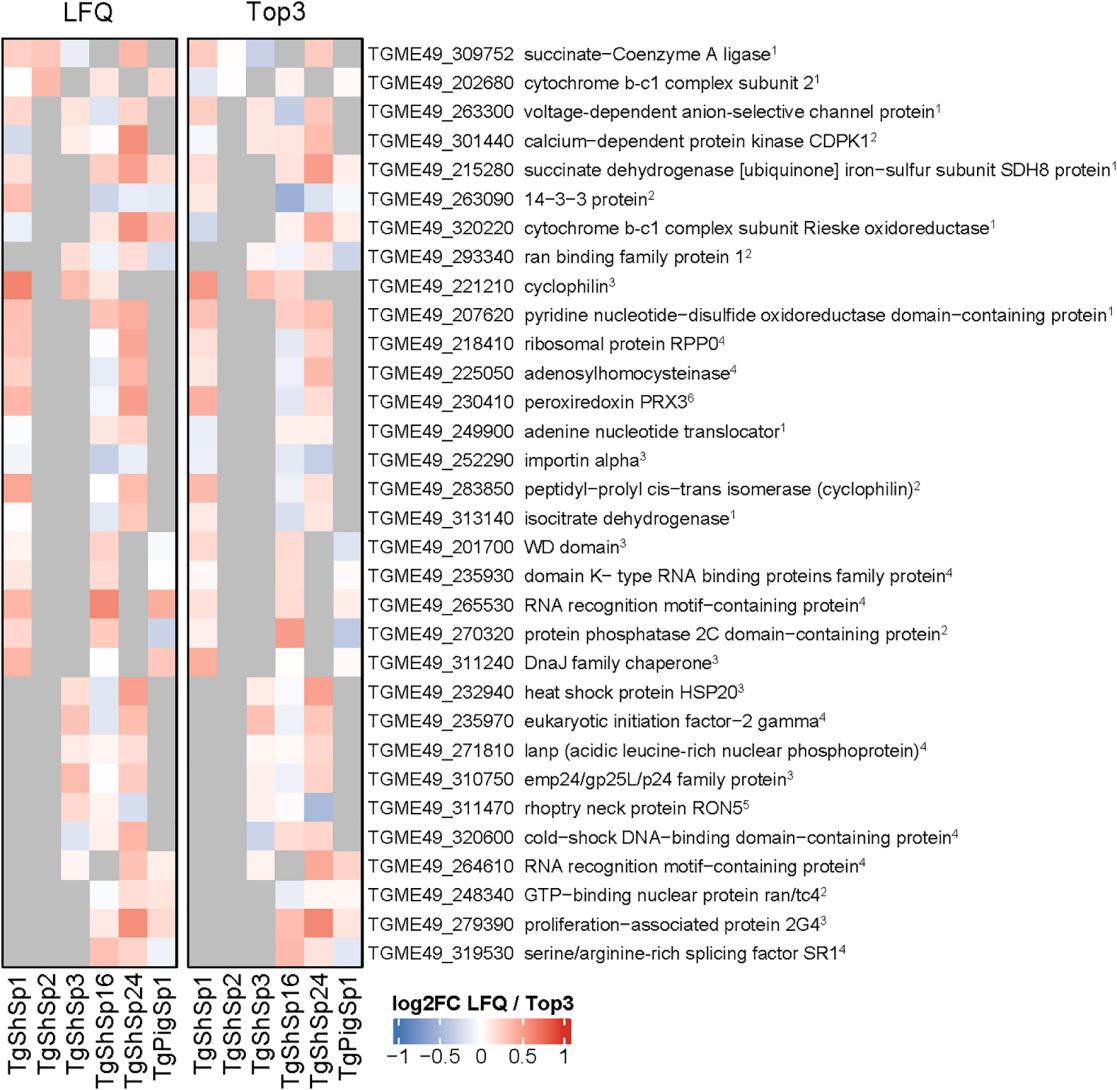
*Toxoplasma gondii* constantly abundant (CA) proteins between high and low passage numbers by both LFQ and TOP3 quantification. Heatmap displaying the log2 fold change (log2FC) values of constantly abundant (CA) proteins (log2FC between -1 and 1, p adjusted < 0.05). Cells in grey indicate proteins that do not meet the criteria for CA proteins between high and low passage number. Principal function for each identified protein is indicated: ^1^citrate cycle, respiration; ^2^signal transduction; ^3^protein modification and processing; ^4^gene expression; ^5^host interaction; ^6^ oxidative stress.

Amongst the 249 proteins identified by equivalence tests as CA between low and high passages, 156 were unique, *i.e.* protein abundance remained unaltered in one isolate only (**Figure 2B**). Of these, 64 were constant in two, 22 in three and 7 in four isolates (**Supplementary Table S3**). None of these proteins were identified as CA in all six isolates. Overall, the highest number of CA proteins, namely 159, was found in TgShSp16, followed by 102 in TgShSp24, 62 in TgShSp1, 33 in TgPigSp1, 20 in TgShSp3 and only 2 in TgShSp2 (**Supplementary Table S3**). According to their respective annotations, the 32 “common” constant proteins presented (among 3 or 4 strains) in **Figure 3** were involved in essential cellular processes, nine of them in gene expression (e.g. translation), eight in mitochondrial energy metabolism, seven in protein modification and processing, and six in signal transduction, with calcium-dependent protein kinase 1 (CDPK1; TGME49_301440) as a prominent example. Protein abundance for several of these proteins is detailed in **Supplementary Figure 1**. This view is reinforced when looking at the complete subset of constant proteins (**Supplementary Table S3**).

In order to comparatively illustrate the variation of protein abundance between low and high passages and among isolates, the quantities of six “variable” proteins (thus neither significant DA nor CA) were specifically illustrated (**Supplementary Figure 2**). These proteins were SRS29B, the major surface protein SAG1 encoded by TGME49_233460, as well as proteins recognized as relevant virulence factors for *T. gondii*: the dense granule proteins GRA12 and GRA25, encoded by TgME49_288650 and TGME49_290700, and the rhoptry proteins ROP18, ROP5 and ROP17, encoded by TGME49_205250, TGME49_308090 and TGME49_258580, respectively. SAG1 and GRA12, followed of GRA25, were remarkably abundant proteins within this subset. Among rhoptry proteins, ROP5 and ROP17 were present in all isolates, whereas, as expected, ROP18 was below the detection level in the Type III strains TgShSp24 and TgPigSp1 (**Supplementary Figure 2**). Of note, ROP proteins, including ROP16 (**Supplementary Table S2**), did not show a clear tendency of variation between low and high passages in both genetic Types of isolates.

### 3.3 Differentially abundant proteins detected in Type II and III canonical isolates are associated with phenotypic changes after adaptation in cell culture

**Figure 4:**
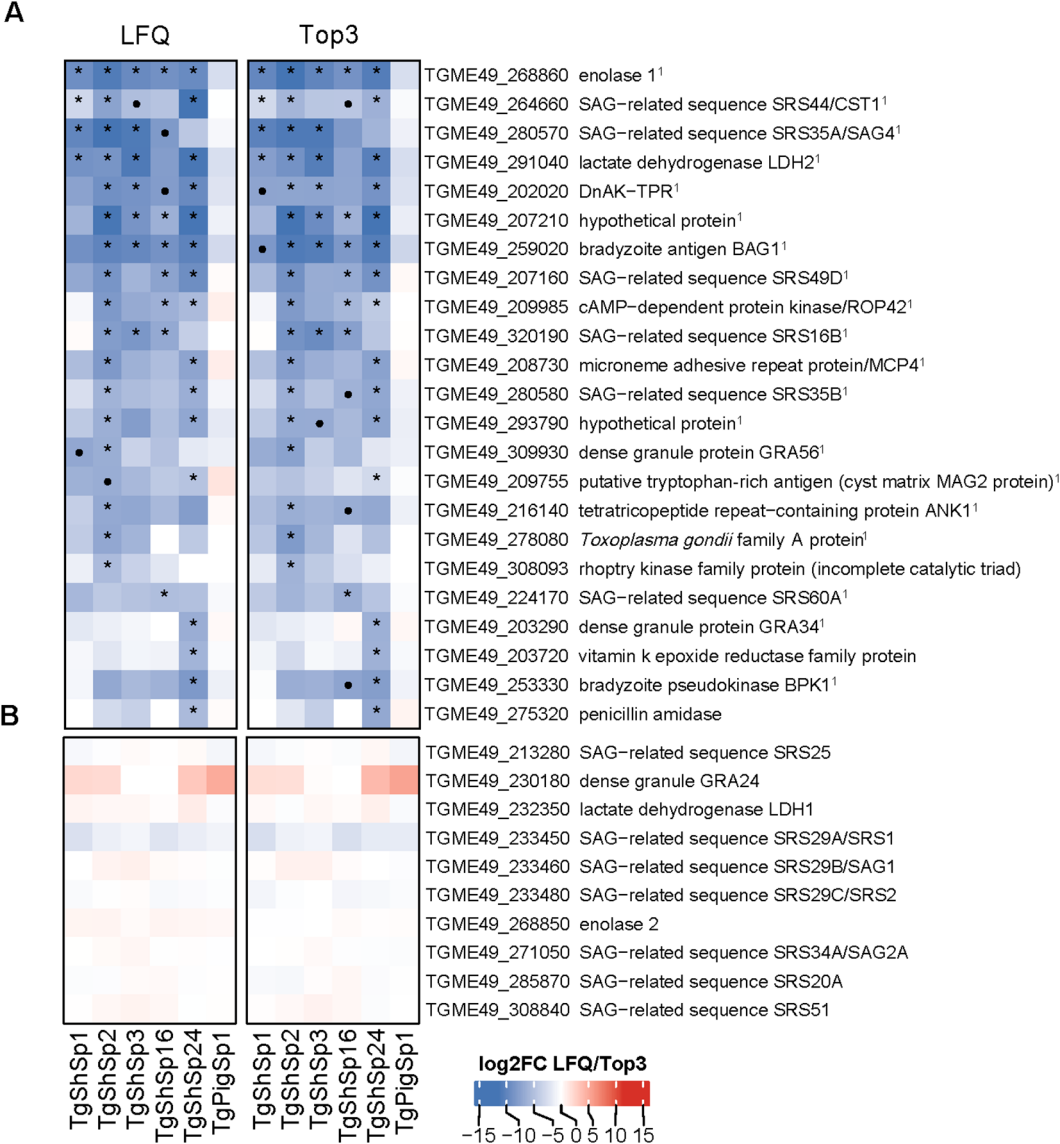
*Toxoplasma gondii* significantly lower differentially abundant (DA) proteins at high *vs*. low passages by LFQ and TOP3. **(A)** Heatmap showing log2 fold change (log2FC) values in low *vs*. high passage comparisons for each isolate. Significantly more DA proteins in low passages (log2FC > 1, p adjusted < 0.05) are marked with an asterisk (*) if consistently identified by both quantification methods: LFQ and Top3, or a dot (•) if specific to only one method. ^1^Indicates bradyzoite-specific proteins. **(B)** Heatmap displaying the log2FC values of tachyzoite-specific proteins. In both heatmaps, log2FC expression levels are indicated in increasing red (high) or blue (low) colors scale.

**Figure 5:**
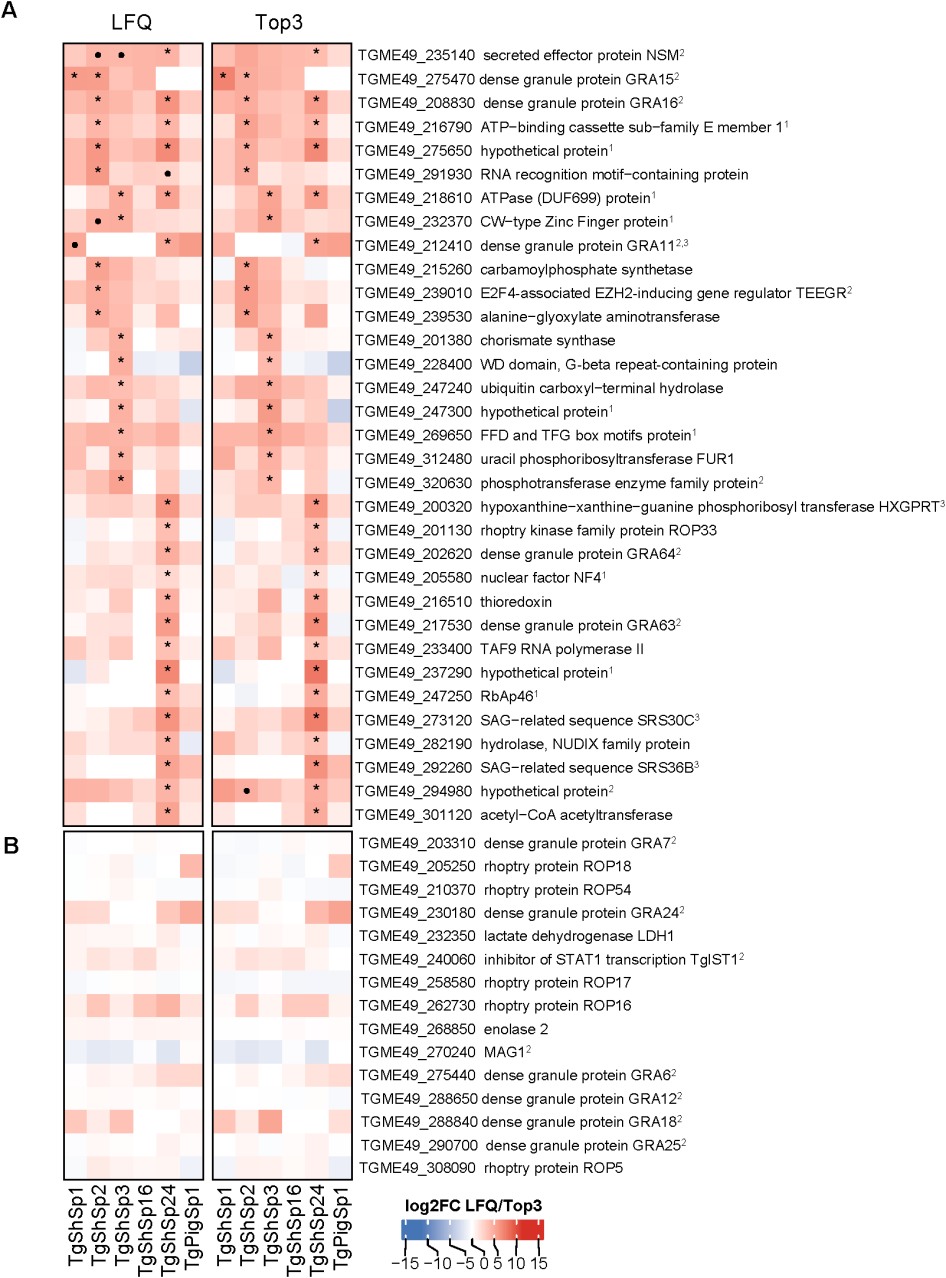
*Toxoplasma gondii* significantly higher differentially abundant (DA) proteins at high *vs*. low passages by LFQ and TOP3. **(A)** Heatmap showing log2 fold change (log2FC) values in low *vs*. high passage comparisons for each isolate. Significantly more DA proteins in high passages (log2FC< -1, p adjusted < 0.05) are marked with an asterisk (*) if consistently identified by both quantification methods: LFQ and Top3, or a dot (•) if specific to only one method. **(B)** Heatmap displaying the log2FC value of *T. gondii* virulent factors without significant variations is shown. In both heatmaps, log2FC expression levels are indicated in increasing red (high) or blue (low) colors scale. ^1^Indicates identified proteins located at the nucleus or nucleolus according to ToxoDB data-hyperLOPIT; ^2^Indicates identified dense granule secreted proteins (GRAs). ^3^Indicates proteins encoded by genes highly expressed in cat early enteric stages (EES) or merozoites (Hehl et al., 2015; Ramakrishnan et al., 2019; Antunes et al., 2024).

DA proteins between low and high passages were detected only in five of the six isolates; DA proteins were not found in TgPigSp1 (**Figures 4**, **5**; full dataset is shown in **Supplementary Table S4**).

#### 3.3.1 Bradyzoite-related proteins exhibit diminished abundance after successive passages in culture

As shown in **Figure 2A**, 23 proteins were abundant at significantly lower levels at high passage. The highest number of proteins that were differentially downregulated at high passages were identified in TgShSp2 (n=17), followed by TgShSp24 (n=16). By contrast, TgShSp3 and TgShSp16 showed 7 proteins with significantly lower levels at high passages, and TgShSp1 only 4 (**Figure 4A**). Most of the diminished (less abundant) proteins at higher passages were specific bradyzoite-related proteins (**Figure 4A**). The most prominent of these DA proteins with lower expression at high passage was enolase 1 (TGME_268860) found in 5 strains, followed by a hypothetical protein encoded by TGME49_207210, the bradyzoite antigen BAG1 (TGME49_259020), and lactate dehydrogenase 2 (LDH2) (TGME49_291040) found as DA proteins in 4 out of 5 isolates. Another prominent marker within this subset of proteins was SRS35A (TGME49_280570), also known as bradyzoite-related surface antigen SAG4 that was practically absent in the Type III strains (TgShSp24 and TgShPigSp1) (**Supplementary Figure S3**), and SRS44 (TGME49_264660), identified as CST1 protein, a well-recognized tissue-cyst marker that showed minor abundance in TgShSp16 and TgPigSp1 isolates (**Supplementary Figure S3**). In TgShSp2 and TgShSp3 isolates, all six bradyzoite markers had significantly lower levels in high *vs.* low passages. However, due to the large standard deviations, these differences were not persistently statistically significant throughout different isolates (**Figure 4**). A larger number of more abundant proteins at low passage was also identified for TgShSp2, TgShSp24 or TgShSp16, specifically bradyzoite and cyst components such as SRS35B (TGME49_280580), BPK1 (TGME49_253330) and GRA56 (TGME49_309930) among others (**Figure 4A**). No differences in abundance of tachyzoite-associated proteins were found, including SAG1 (**Figure 4B**; **Supplementary Figure 2A**).

In agreement with these results, formation of “mature” cysts, *i.e.* cysts resistant to the pressure of needle passage during sample collection/harvesting, were identified in those samples originating from isolates at low passages maintained in cell culture for more than two days (**Supplementary Table S1**). The formation of mature cysts at low passage number was also confirmed by DBL-staining (**Supplementary Table S1**; **Supplementary Figure S4**). Mature cysts were not found in any sample from isolates maintained at high passage, and not in the Type II TgShSp16 and Type III TgPigSp1 examined at low and high passages, which showed minor abundances of bradyzoite components at low passages (**Figure 4A**).

#### 3.3.2 Increased DA proteins at high passages are associated with exacerbation of virulence in mice

As mentioned above (see **Figure 2A**) and as presented in the complete dataset of DA proteins (**Supplementary Table S4**), 33 proteins were significantly more abundant at high *vs.* low passages (**Figure 3A**). Among them, 28 were unique DA proteins that were identified only in one isolate, originating unique profiles: twenty DA proteins were identified in TgShSp24, followed by nine proteins in TgShSp3 and seven in TgShSp2 (**Figure 3A**). Interestingly, these isolates showed exacerbation of virulence in mice after 40 passages *in vitro*, increasing morbidity in TgShSp2 and TgShSp3 and mortality in TgShSp24 (**Table 1**). Notably, no DA and only one DA protein was detected at high passage numbers in TgShSp16 and TgShSp1, respectively, the only isolates showing attenuation or no changes in an already low level of virulence in mice after *in vitro* culture adaptation. Similarly, no DA proteins were found in the Type III TgPigSp1 isolate, which showed to be the most virulent one in mice, with no drastic variation, at both low and high passages.

Five proteins associated with elements located in the parasite nucleus and in the dense granules were found in 2 isolates to have significantly increased expression at high passage. Two proteins with homologies to the ATP-binding cassette sub-family E member 1 encoded by TGME49_216790, the ATP-ase homolog encoded by TGME49_218610, and a hypothetical protein encoded by TGME49_275650, were more abundant at high passages in Type II (TgShSp2 and TgShSp3) and Type III (TgShSp24) isolates. A CW-type Zinc Finger protein encoded by TGME49_232370 was also increased in abundance at high passages for Type II TgShSp3, and with a tendency to be increased in TgShSp2 (**Figure 5A**; **Supplementary Figure S5**). All these proteins were located in the nucleus or nucleolus, as it was the case for the proteins that increased in TgShSp3 and TgShSp24 (**Figure 5A**). A subset of GRA proteins was also abundant at higher levels at high passage numbers of these Type II and Type III isolates (**Figure 5A**; **Supplementary Figure S6**). GRA15 (TGME49_275470), GRA16 (TGME49_208830) and E2F4-associated EZH2-inducing gene regulator (TEEGR, TGME49_239010) were increased in Type II TgShSp2. Expression of GRA15 was below the detection limit at low passages and also significantly increased at high passages for Type II TgShSp1. The quantities of these proteins in Type II TgShSp16 were too small or had a too large standard deviation to allow detection of significant differences between low and high passages (**Supplementary Figure S6**). As expected, GRA15 was not detected in the Type III isolates TgShSp24 and TgShPigSp1 (**Figure 5A**; **Supplementary Figure S6**). A similar profile was observed for TEEGR in Type II isolates, although no differences were detected between passages in TgShSp1 (**Supplementary Figure S6**). Levels of GRA16 (TGME49_208830), GRA64 (TGME49_202620) and GRA63 (TGME49_217530) were clearly increased in the Type III TgShSp24 isolate at high passages (barely undetectable at low passages), with similar levels to those observed in the Type III TgPigSp1 isolate at both low and high passages (**Supplementary Figure S6**). The secreted effector NSM encoded by TGME49_235140 was significantly increased in Type III TgShSp24, and with a tendency to be more abundant at high passages in TgShSp2 and TgShSp3 (**Supplementary Figure S6**).

In addition, GRA11A (TGME49_212410) and SRS36B (TGME49_292260) were strongly increased at high levels in the Type III TgShSp24 isolate. GRA11B and SRS36B were also increased at high passages in the other Type III isolate TgPigSp1 isolate, although not significance or a tendency to significance was found (**Figure 5A**; **Supplementary Figure S7**). Among Type II isolates, solely GRA11A in Type II TgShSp1 showed a tendency to be more abundant at high passages (**Supplementary Figure S7**).

By contrast, no change in protein abundance was observed for other GRAs, ROPs or SRSs (**Figure 5B**; **Supplementary Figure S2**).

### 3.4 Overall proteome correlation analyses confirm variation after adaptation related to *in vitro* and *in vivo* phenotypic changes

**Figure 6:**
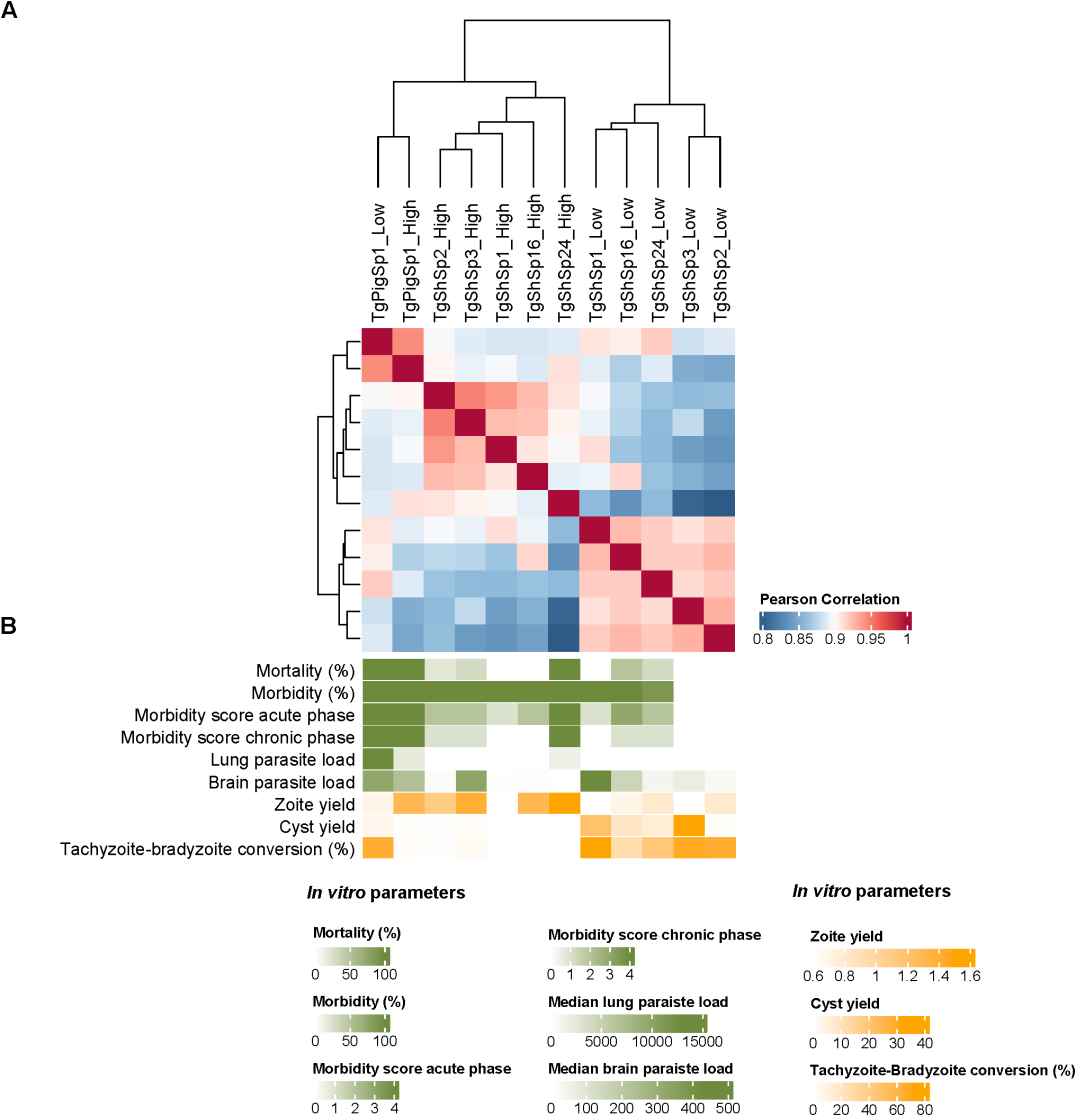
Correlation of *Toxoplasma gondii* proteomes. **(A)** Heatmap showing the Pearson correlation analyses among *T. gondii* proteomes at high *vs.* low passages. **(B)** Graduation of phenotypic traits determined for *T. gondii* isolates at high and low passages in mice (green color gradient) and *in vitro* (yellow color gradient).

Pearson correlation analyses demonstrated close proximity among the six isolates proteomes (r > 0.8), as expected. Notwithstanding Pearson correlation analyses also demonstrated clustering with a clear segregation of proteomes determined by low and high passages for those *T. gondii* isolates that showed DA proteins. TgPigSp1 without DA between low and high passages clustered together with proteomes at high passage from the other isolates, although it was secondly segregated from these proteomes showing the highest r (**Figure 6A**). Even though there were no statistical significance, proteome segregation was apparently associated with variations in *in vitro* and *in vivo* phenotypic traits (**Figure 6B**). *Toxoplasma gondii* isolates not causing mortality and limited morbidity levels in mice at low passages (TgShSp1, TgShSp2 and TgShSp3) showed the highest capacities for tachyzoite-to-bradyzoite conversion and spontaneously cyst formation, together with low tachyzoite production *in vitro*. On the other hand *T. gondii* isolates at high passages with a 100% of mouse mortality (TgShSp24 and TgPigSp1) showed the highest parasite loads in lungs, in agreement with their proliferation capacities and diminished cyst production *in vitro*. The TgShSp1, TgShSp2 and TgShSp16 isolates at high passages with limited mortality levels in mice (0-20%) also showed the lowest parasite loads in the lungs and also in brain, which could be likely associated with a lower *in vitro* cyst production after adaptation.

## 4 Discussion

Despite the differences in the genetic background and origin of the six *T. gondii* strains investigated in our study, only minor differences were observed when comparing their respective proteomes at low and high passage numbers. The limited variation in protein abundance was not unexpected due to the short time frame of *in vitro* culture (approximately 40 passages), but this adaptation period was enough to observe some relevant differences. These differences are validated by the fact that some proteins previously shown to be expressed only in Type II but not in Type III strains such as GRA15 (Merritt et al., 2024; Rosowski et al., 2011) and ROP18 (Saeij et al., 2006), were indeed undetectable in our Type III strains but highly abundant in the Type II strains. Similar differences, especially a decreased abundance of GRA proteins detected by less resolute two-dimensional polyacrylamide gel electrophoresis and mass spectrophotometry, were noted previously in a Type I isolate that was attenuated upon prolonged *in vitro* culture (Nischik et al., 2001).

In our study, properly identified DA proteins were associated with the phenotypic changes observed earlier following culture in Vero cells (Colos-Arango et al., 2023). Correlation analyses demonstrated the clear segregation across *T. gondii* proteomes with DA proteins after *in vitro* adaptation. Moreover, in agreement with correlations determined in a previous study (Colos-Arango et al., 2023), an association among virulence in mice with loss of cyst formation and increased tachyzoite yield *in vitro*, before and after adaptation to cell culture, was also found in proteome segregation. After 40 passages, all isolates showed a drastic reduction in the expression of bradyzoite-related proteins, as expected from our previous phenotypic observations demonstrating a decrease in the spontaneous tachyzoite-to-bradyzoite conversion abilities at higher passage numbers (Colos-Arango et al., 2023). The diminished abundance of bradyzoite-specific markers at high passages was the most evident shared pattern in our Type II and Type III canonical isolates, with TgPigSp1 as the only exception. This Type III isolate maintained the capacity to form cysts after culture adaptation, although it only developed mature cysts sporadically (Colos-Arango et al., 2023). In fact, TgPigSp1 demonstrated the lowest levels of bradyzoite-related proteins, remaining invariable after increased passage numbers, and a DBL-positive cyst wall could not be detected at any time. Recently, we proposed a “propensity stage” model for cyst formation in recently obtained canonical *T. gondii* isolates, since they can produce cysts spontaneously in the absence of stress conditions in immortalized cells such as Vero.

Interestingly, some particular proteins such as GRA11A and SRS36B, encoded by genes highly expressed in *T. gondii* entero-epithelial stages, such as the merozoite stage, were found to be increasingly abundant at high passage numbers in the Type III TgShSp24 and Type II TgShSp1 strains (Antunes et al., 2024; Ramakrishnan et al., 2019; Hehl et al., 2015). This event occurs naturally only in felid enterocytes when tissue cysts containing bradyzoites are ingested.

Leaving aside the common diminished abundance for bradyzoite-related proteins at high passage numbers in all isolates, there was no common pattern of significantly more abundant proteins, with practically the presence of individual profiles for each isolate and only 4 proteins being unique in 2 of the isolates. Interestingly, no significant increase in protein abundance, or only limited variation, was detected for TgShSp1 and TgShSp16 isolates in low *vs.* high passages, with no changes or attenuation of virulence (morbidity/mortality), respectively. In addition, no changes in protein abundance were detected in the highly virulent TgPigSp1 isolate, which had also not shown any changes in virulence after culture adaptation (100% mortality at both low and high passages, Colos-Arango et al., 2023). Therefore, DA proteins were mainly studied with the aim of unravelling virulence changes observed in TgShSp2, TgShSp3 and TgShSp24 isolates.

According to hyperplexed localization of organelle proteins by isotope tagging (HyperLOPIT) studies (Barylyuk et al., 2020), the commonly increased proteins ATP-binding cassette sub-family E member 1 encoded by TGME49_216790 and the hypothetical protein encoded by TGME49_275650 are localized in the nucleus, and the ATPase encoded by TGME49_218610 in the nucleolus. The CW-type Zinc Finger protein encoded by TGME49_232370 and predicted to be located in nucleolus was also significantly increased in TgShSp3, and with a tendency for TgShSp2 isolate. Although the exact role played by these proteins has not been studied in *T. gondii*, they could be involved in chromatin remodeling and epigenetic regulation (Farhat et al., 2020). Hence, the upregulation of these proteins after prolonged *in vitro* culture may be correlated to phenotypic changes such as a higher proliferation rate at higher passages for TgShSp2 or TgShSp3, and TgShSp24 isolates. In our previous studies we observed that the loss of spontaneous cyst formation after successive passages is accompanied by a faster growth rate. However, a higher proliferation alone is not enough to account for the changes in virulence observed in murine models, especially for the Type II TgShSp1 and TgShSp16 isolates, which increased their multiplication rate and zoite production *in vitro* but showed no changes in mortality in mice (Colos-Arango et al., 2023), as occurred in previous studies performed with the Type I BK isolate (Nischik et al., 2001).

Notably, we detected an increased abundance of several GRA and ROP proteins at high passages. These effectors are well known for their role in co-opting the host cell response. For instance, the ROP5/ROP17/ROP18 complex phosphorylates the immune related GTPases (IRG), thus preventing an effective coating of the parasitophorous vacuole membrane (PVM) which in turns leads to an increased resistance to IFN-γ-mediated killing (Mukhopadhyay et al., 2020). We did not observe a significant variation in the abundance of these proteins among the Type II or III isolates used in our study. This was expected, since only Type I strains have an active combination of all three components of the ROP5/ROP17/ROP18 complex (Mukhopadhyay et al., 2020). Another important effector is ROP16, which has been shown to induce a sustained activation of the host transcription factors STAT3 and STAT6, in turn leading to the dampening of Th1 inflammatory responses (Jensen et al., 2013). ROP16 was also not variable among isolates and culture maintenance in our study. Regarding dense granule proteins, TgNSM abundance was significantly increased in those isolates that showed increased virulence at high passage (TgShSp24) and had a tendency to be more abundant in TgShSp2 and TgShSp3. The dense granule secreted TgNSM effector is exported to the host-cell nucleus and has been recently described to increase levels of the NCoR/SMRT complex, a repressor of various transcription factors that inhibit IFN-regulated genes involved in cell necroptotic death, ultimately protecting the parasite’s intracellular niche (Rosenberg and Sibley, 2021). Similarly, the dense granule-resident effector TEEGR/HCE1 is exported into the host cell nucleus to epigenetic silencing of a subset of NF-κB-regulated cytokines, thereby strongly contributing to the host immune equilibrium that promotes parasite persistence in mice (Braun et al., 2019). TEEGR abundance was increased at high passages in all of our Type II and III strains, although a statistically significant difference was only detected for TgShSp2. This suggests that this effector might be related to the higher proliferation of tachyzoites observed *in vitro* at high passages. Moreover, TEEGR directly counteracts the nuclear factor-κB (NF-κB) signaling pathway activated by GRA15 in Type II isolates, which leads to the release of pro-inflammatory cytokines such as IL-12, TNF, and IL-1β from infected cells (Ihara et al., 2020). Hence, it is tempting to hypothesize that the increased abundance of TEEGR at high passages may be counteracting the activity of GRA15 in the clonal Type II TgShSp2 isolate, in turn accounting for the exacerbation of virulence observed in mice for this strain (Colos-Arango et al., 2023). Conversely, TEEGR was not significantly increased in TgShSp1, which showed a marked increase of GRA15 abundance at high passage and maintained a low virulence profile regardless of the passage number (Colos-Arango et al., 2023). GRA16 is also exported to the host cell nucleus, where it increases the p53 tumor suppressor levels, generating a pro-apoptotic state in the infected cell (Henry et al., 2024; Bougdour et al., 2013). GRA16 is well conserved among I, II and III clonal Types, and it is considered a virulence factor, since Type II-PRU strains that are deficient in GRA16 exhibit attenuated virulence (Bougdour et al., 2013). In our study, GRA16 showed a significantly increased abundance at high passages in TgShSp2 and TgShSp24 isolates. Remarkably, all identified GRA proteins were maintained at low levels of abundance at both low and high passages in the TgShSp16 isolate, which showed an attenuation in virulence in mice after *in vitro* culture adaptation (Colos-Arango et al., 2023). Additional GRA proteins such as GRA63 and GRA64 were increased in Type III TgShSp24 at high passage numbers. Both GRA63 and 64 are localized to the PVM, being partially exposed to the host cell cytoplasm (Mayoral et al., 2022; Cygan et al., 2021). These two GRA proteins appear to interact with components of the host endosomal sorting complexes required for transport (ESCRT), which mediates many functions in the host cell related to membrane remodeling, many of which could be relevant to *T. gondii* lytic cycle but apparently dispensable *in vivo*, at least for Type II strains (Mayoral et al., 2022; Cygan et al., 2021). The potential role of GRA63 and GRA64 in a GRA15-lacking background, such as that present in Type III strains, remains to be assessed. Finally, it is worth mentioning that our results were obtained in Vero cell cultures, an interferon-deficient cell line where no immune pressure is present (Osada et al., 2014).

CA proteins constitute the by far biggest proteome subset found in our study, showing no significantly altered abundance levels and representing one tenth of the proteome obtained from our *T. gondii* isolates. These CA proteins may be regarded as valuable “reference proteins” for protein quantification, e.g. in proteomic analyses or quantitative immunoblot experiments. Although we referred to these proteins as constantly abundant by analogy to DA proteins (differentially abundant), the levels of their corresponding mRNA expression may vary. Therefore, parallel quantitative investigations of the proteome and the transcriptome with respect to the same ORFs are warranted to provide further insights.

Regardless, these CA proteins could represent interesting vaccine or drug targets. Examples of *T. gondii* vaccine candidates identified herein are proteins involved in host-parasite interactions such as RON4 and RON5 (Zhao et al., 2016; Zhang et al., 2015). Another CA protein identified in three of the six strains was cyclophilin, which is considered an immunomodulator involved in mediating the host-pathology by inducing an inflammatory response during infection (Gong et al., 2013; Yu et al., 2013). Moreover, *T. gondii* is known to be susceptible to high doses of cyclosporin A, a cyclophilin ligand *in vitro* (Mack and McLeod, 1984), and also *in vivo* at concentrations not suppressing the host immune response (McCabe et al., 1986). One of the most abundantly expressed *T. gondii* proteins, the surface antigen SAG1 or SRS29B, a valid antigen for establishing subunit and vector-based vaccine models as shown during the last two decades (Imhof et al., 2022; Seng et al., 2004; Haumont et al., 2000), was not within the subset of CA proteins identified herein by equivalence test, but was found to be “variable”, *i.e.* not exhibiting DA but also not being CA.

With respect to potential drug targets, it is not surprising to find proteins involved in gene expression and mitochondrial energy metabolism. While the first-line treatments of toxoplasmosis consist of pyrimethamine-sulfadiazine or trimethoprim-sulfamethoxazole that interfere in the folic acid pathway, other treatment options include the macrolide antibiotic spiramycin (early in pregnancy) or the lincosamide clindamycin, both of which inhibit translation upon binding to ribosomes (Alday and Doggett, 2017). Atovaquone, a naphthoquinone, is another treatment option that inhibits a wide range of apicomplexan protozoans including *Plasmodium* spp., most likely by acting as an antagonist of ubiquinone (Saleh et al., 2007). Consequently, atovaquone interferes in the electron transfer from the succinate dehydrogenase (complex II) via ubiquinone to cytochrome c by the cytochrome bc1 complex (complex III). The same accounts for the quinolone decoquinate and particularly for the related endochin-like quinolones (Doggett et al., 2012). Members of both complex II and III are constitutively expressed in four of the six evaluated strains. Another prominent target, the calcium-dependent kinase 1 (CDPK1; TGME49_301440) is one of the CA proteins in four of the strains. TgCDPK1 is essential for motility, adhesion to host cells, invasion and egress, as shown by inhibitor studies (Kieschnick et al., 2001; Lourido et al., 2012). Specific drugs called “bumped kinase inhibitors” (BKIs) are effective against many apicomplexan parasites of medical and veterinary relevance including *Cryptosporidium parvum* (Hulverson et al., 2017; Ojo et al., 2014), *T. gondii* (Sánchez-Sánchez et al., 2024), *Cystoisospora suis* (Shrestha et al., 2019), *Sarcocystis neurona* (Ojo et al., 2016), *Besnoitia besnoiti* (Jiménez-Meléndez et al., 2017) and *Neospora caninum in vitro* and *in vivo* (Van Voorhis et al., 2021; Ojo et al., 2014). However, besides CDPK1 other drug targets could be involved in the mechanisms of action of BKIs in these parasites (Imhof et al., 2024).

It can be safely hypothesized that CA proteins maintained within a narrow range of variation may be essential, and that the subset of constant proteins presented here may stimulate the search for novel targets.

From a practical point of view, the isolation of novel *T. gondii* strains from infected animals or humans is surely rewarding in terms of broadening the view on “patho-biodiversity”. However, the work with established laboratory strains focusing on common, rather than diverse proteomes, certainly prevails. The identification of common constant proteins using equivalence tests has confirmed well-known drug or vaccine targets, and also suggested potentially novel investigative targets for intervention, as well as proteins involved in metabolic or developmental regulations, all of which can be safely investigated using standard strains such as ME49, using established *in vitro* methodologies and animal models. *Toxoplasma gondii* reference laboratory isolates, as standardized models, had offered undeniable advantages, such as stability in culture (Müller and Hemphill, 2012), usefulness for *in vitro* and *in vivo* test systems to identify novel drugs (Müller and Hemphill, 2024), and the availability of well-established molecular genetic tools (Meissner et al., 2007), including support by a proficient and well-organized database (www.toxodb.org). Moreover, the availability of reference strains worldwide is a prerequisite for replication and reproducibility of results among different research groups, representing the cornerstone of any empirical scientific approach. On the other hand, normalized models based on laboratory-adapted isolates require the application of stress conditions such as alkaline pH in well-established cell lines such as Vero, a fact that may alter the physiological conditions required for the use of drugs against the bradyzoite stage. By contrast, the use of well-developed models based on recently obtained isolates showing a high capacity of spontaneous cyst production, such as in TgShSp1 and TgShSp3, could be a valuable alternative. Thus, further *in vitro* studies aiming at elucidating the mechanisms of spontaneous and induced cyst formation will be paramount.

Finally, thinking outside the box, investigating constant proteins as detailed above within the proteomes of various eukaryote model systems may provide the means to define a minimal eukaryote proteome. This would extend the knowledge obtained from the minimal genome experiments producing artificial prokaryote-like life forms (Gibson et al., 2008a; 2008b) in order to generate artificial eukaryote cells.

## 5 Conclusion

*In vitro* maintenance of recently obtained *T. gondii* isolates can entails changes in the proteome that results in phenotype variation: loss of capacities to tachyzoite-bradyzoite conversion and increased mice virulence. Very interestingly, this study provides evidence of dense granule proteins to be relevant for exacerbation of *T. gondii* virulence after fast *in vitro* adaptation. On the other hand, proteins that are produced invariable constitute potential drug and vaccine targets.

## Data Availability

The datasets presented in this study can be found in online repositories. The names of the repository/repositories and accession number(s) can be found in the article/**Supplementary Material**. The mass spectrometry proteomics data have been deposited to the ProteomeXchange Consortium via the PRIDE (Pérez-Riverol et al., 2025) partner repository with the dataset identifier PXD067210.

## Supplementary material

Supplementary Table 1 Data on sample collection of *T. gondii* Type II and Type III canonical isolates for proteomic analyses and cyst detection.

Supplementary Table 2 Full proteome dataset of the six Type II and Type III canonical *T. gondii* strains.

Supplementary Table 3 Constantly abundant (CA) proteins in the six Type II and Type III canonical *T. gondii* strains as identified by equivalence tests described in Materials and methods section.

Supplementary Table 4 Differentially abundant (DA) proteins in the six Type II and Type III canonical *T. gondii* strains as identified by empirical Bayes tests described in Materials and methods section.

Supplementary Figure 1 Protein abundance of constantly abundant (CA) proteins at high *vs.* low passages in Type II and Type III canonical isolates: CDPK1 **(A)**, DnaJ family chaperone **(B)**, eukaryotic porin protein **(C)**, ubiquinol cytochrome c oxidoreductase **(D)**, ran binding family protein 1 **(E)**, and succinate dehydrogenase Ion-sulfur protein **(F)**. Columns represent the mean, and bars the standard deviation, of label-free quantification (LFQ) values for three biological replicates for each isolate at low (p10) or high (p50) passages (see legend, p10 = p10-p16; p50 = p50-p53). Note that LFQ abundance scale in y-axis varies among charts for easier visualization.

Supplementary Figure 2 Quantification of six “variable” proteins involved in tachyzoite-host interactions in Type II and Type III canonical isolates: SAG1 **(A)**, GRA12 **(B)**, GRA25 **(C)**, ROP18 **(D)**, ROP5 **(E)** and ROP17 **(F)**. Columns represent the mean, and bars the standard deviation, of label-free quantification (LFQ) values for three biological replicates for each isolate at low (p10) or high (p50) passages (see legend, p10 = p10-p16; p50 = p50-p53). Note that LFQ abundance scale in y-axis varies among charts for easier visualization.

Supplementary Figure 3 Quantification of six bradyzoite- and tissue cyst-related differentially abundant (DA) proteins showing significantly lower levels at high *vs.* low passages among the Type II and Type III canonical isolates: ENO1 **(A)**, hypothetical protein TGME49_207210 **(B)**, BAG1 **(C)**, LDH2 **(D)** and CST1 **(E)**. Columns represent the mean, and bars the standard deviation, of label-free quantification (LFQ) values for three biological replicates for each isolate at low (p10) or high (p50) passages (see legend, p10 = p10-p16; p50 = p50-p53). * indicates significant DA proteins by iLFQ and iTOP3 levels (log2 fold-change < -1 or >1; p adjusted < 0.05). Note that LFQ abundance scale in y-axis varies among charts for easier visualization.

Supplementary Figure 4 Immunofluorescence pictures of mature cysts in the Type II isolates TgShSp1 **(A)**, TgShSp3 **(B)** and TgShSp2 **(C)**, and Type III TgShSp24 **(D)** at low passage, stained with FITC-conjugated *Dolichos biflorus* lectin. The scale bar indicates the size in µm.

Supplementary Figure 5 Quantification of four differentially abundant (DA) proteins with significantly higher levels at high *vs.* low passages located at parasite nucleus/nucleolus in Type II and Type III canonical isolates: ATP-binding cassette sub-family E member 1 **(A)**, ATPase (DUF699) protein **(B)**, hypothetical protein TGME49_275650 **(C)** and CW-type Zinc Finger protein **(D)**. Columns represent the mean, and bars the standard deviation of label-free quantification (LFQ) values for three biological replicates for each isolate at low (p10) or high (p50) passages (see legend, p10 = p10-p16; p50 = p50-p53). * indicates significant DA proteins by iLFQ and iTOP3 levels. ^δ^ indicates significant DA protein by only either iLFQ or iTOP3 levels (log2 fold-change < -1 or >1; p-adjusted < 0.05). Note that LFQ abundance scale in y-axis varies among charts for easier visualization.

Supplementary Figure 6 Quantification of six differentially abundant (DA) proteins with significantly higher levels at high *vs.* low passages secreted from dense granules in Type II and Type III canonical isolates: GRA15 **(A)**, GRA16 **(B)**, E2F4-associated EZH2-inducing gene regulator TEEGR **(C)**, secreted effector protein NSM **(D)**, GRA64 **(E)** and GRA63 **(F)**. Columns represent the mean, and bars the standard deviation of label-free quantification (LFQ) values for three biological replicates for each isolate at low (p10) or high (p50) passages (see legend, p10 = p10-p16; p50 = p50-p53). * indicates significant DA proteins by iLFQ and iTOP3 levels. ^δ^ indicates significant DA protein by only either iLFQ or iTOP3 levels (log_2_ fold-change < -1 or >1; p-adjusted < 0.05). Note that LFQ abundance scale in y-axis varies among charts for easier visualization.

Supplementary Figure 7 Quantification of two differentially abundant (DA) proteins with significantly higher levels at high *vs.* low passages in the Type III TgSpSp24 isolate related to merozoite stage: GRA11A **(A)** and SRS36B **(B)**. Columns represent the mean, and bars the standard deviation of label-free quantification (LFQ) values for three biological replicates for each isolate at low (p10) or high (p50) passages (see legend, p10 = p10-p16; p50 = p50-p53). * indicates significant DA proteins by iLFQ and iTOP3 levels. ^δ^ indicates significant DA protein by only either iLFQ or iTOP3 levels. Note that LFQ abundance scale in y-axis varies among charts for easier visualization.
