# Supplementary figures and images for: Proteome changes during in vitro culture adaptation of Toxoplasma gondii archetypal II and III field isolates
Source: Front Cell Infect Microbiol. 2025 Sep 16;15:1633384. doi: 10.3389/fcimb.2025.1633384 (PMC12479500; doi:10.3389/fcimb.2025.1633384)

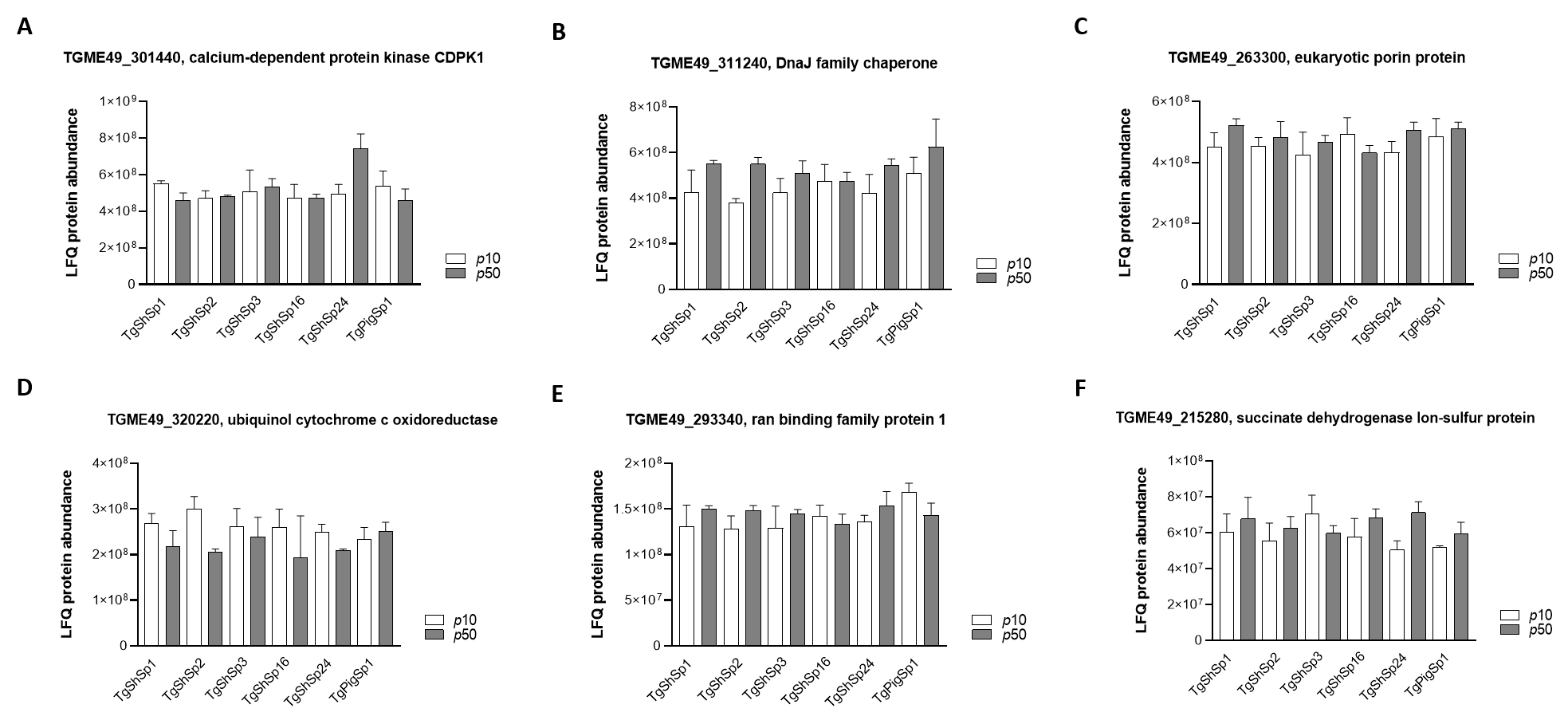

Supplement: Supplementary Figure 1 — Protein abundance of constantly abundant (CA) proteins at high vs. low passages in Type II and Type III canonical isolates: CDPK1 (A), DnaJ family chaperone (B), eukaryotic porin protein (C), ubiquinol cytochrome c oxidoreductase (D), ran binding family protein 1 (E), and succinate dehydrogenase Ion-sulfur protein (F). Columns represent the mean, and bars the standard deviation, of label-free quantification (LFQ) values for three biological replicates for each isolate at low (p10) or high (p50) passages (see legend, p10 = p10-p16; p50 = p50-p53). Note that LFQ abundance scale in y-axis varies among charts for easier visualization. [file Image1.tif]

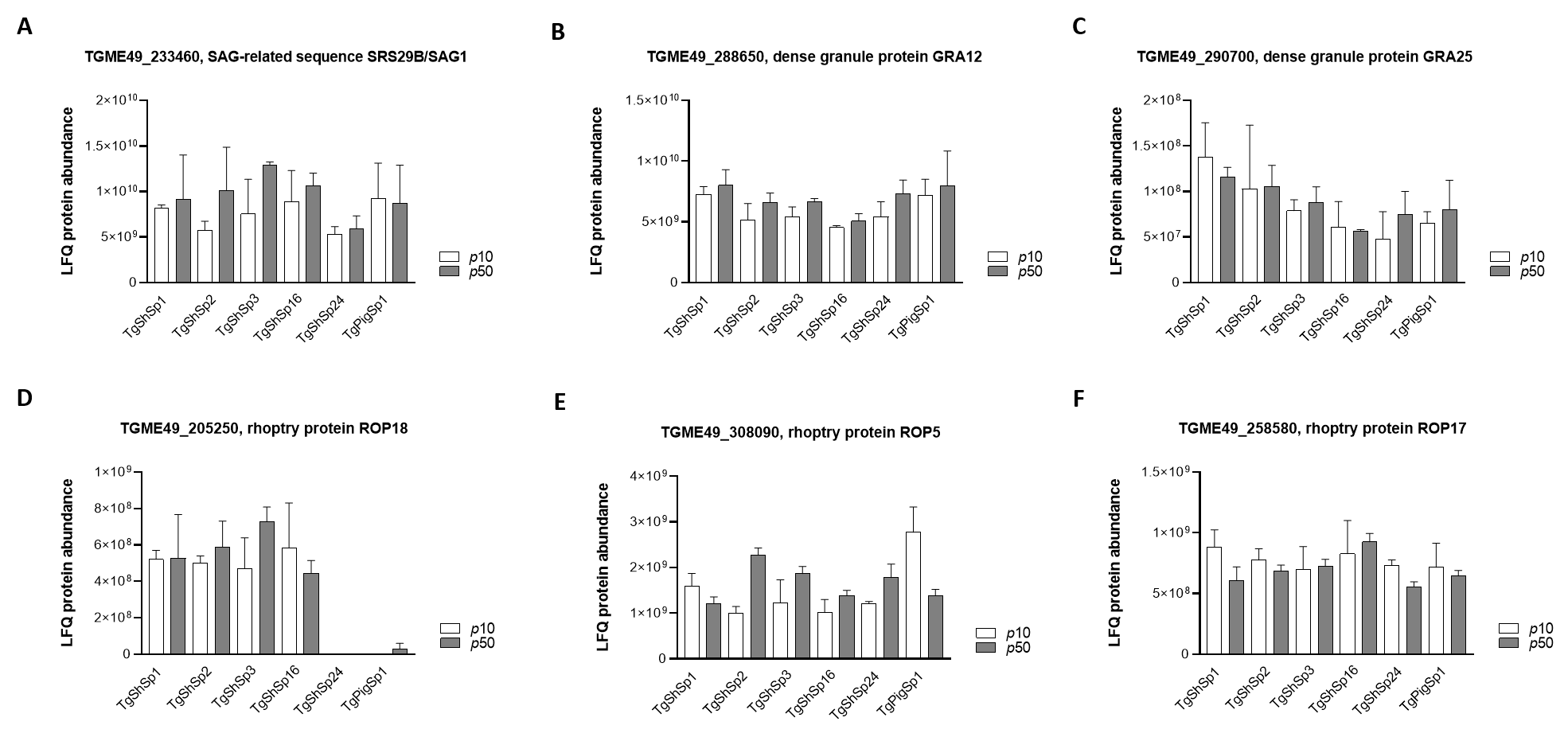

Supplement: Supplementary Figure 2 — Quantification of six “variable” proteins involved in tachyzoite-host interactions in Type II and Type III canonical isolates: SAG1 (A), GRA12 (B), GRA25 (C), ROP18 (D), ROP5 (E) and ROP17 (F). Columns represent the mean, and bars the standard deviation, of label-free quantification (LFQ) values for three biological replicates for each isolate at low (p10) or high (p50) passages (see legend, p10 = p10-p16; p50 = p50-p53). Note that LFQ abundance scale in y-axis varies among charts for easier visualization. [file Image2.tif]

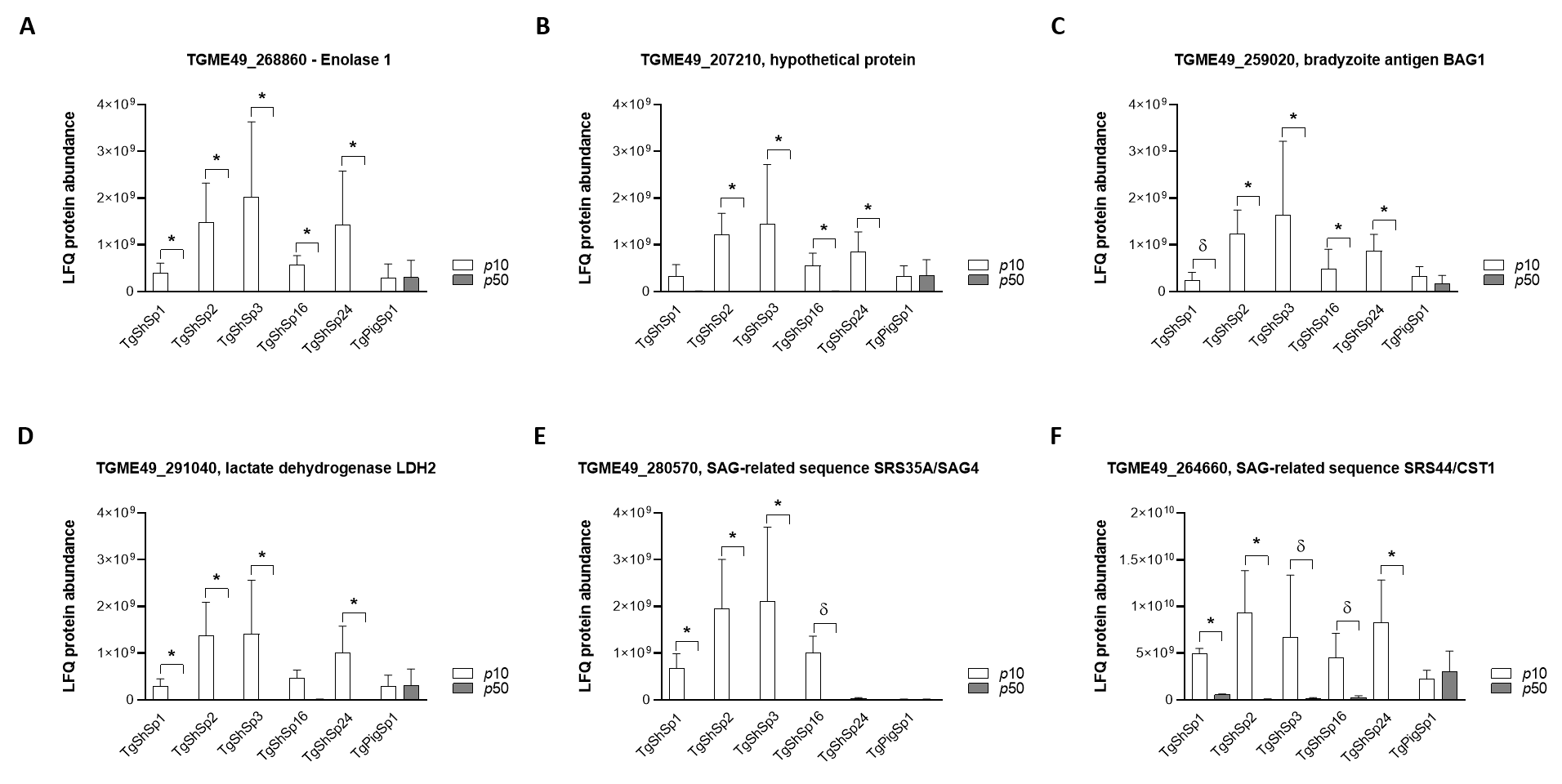

Supplement: Supplementary Figure 3 — Quantification of six bradyzoite- and tissue cyst-related differentially abundant (DA) proteins showing significantly lower levels at high vs. low passages among the Type II and Type III canonical isolates: ENO1 (A), hypothetical protein TGME49_207210 (B), BAG1 (C), LDH2 (D) and CST1 (E). Columns represent the mean, and bars the standard deviation, of label-free quantification (LFQ) values for three biological replicates for each isolate at low (p10) or high (p50) passages (see legend, p10 = p10-p16; p50 = p50-p53). * indicates significant DA proteins by iLFQ and iTOP3 levels (log2 fold-change < -1 or >1; p adjusted < 0.05). Note that LFQ abundance scale in y-axis varies among charts for easier visualization. [file Image3.tif]

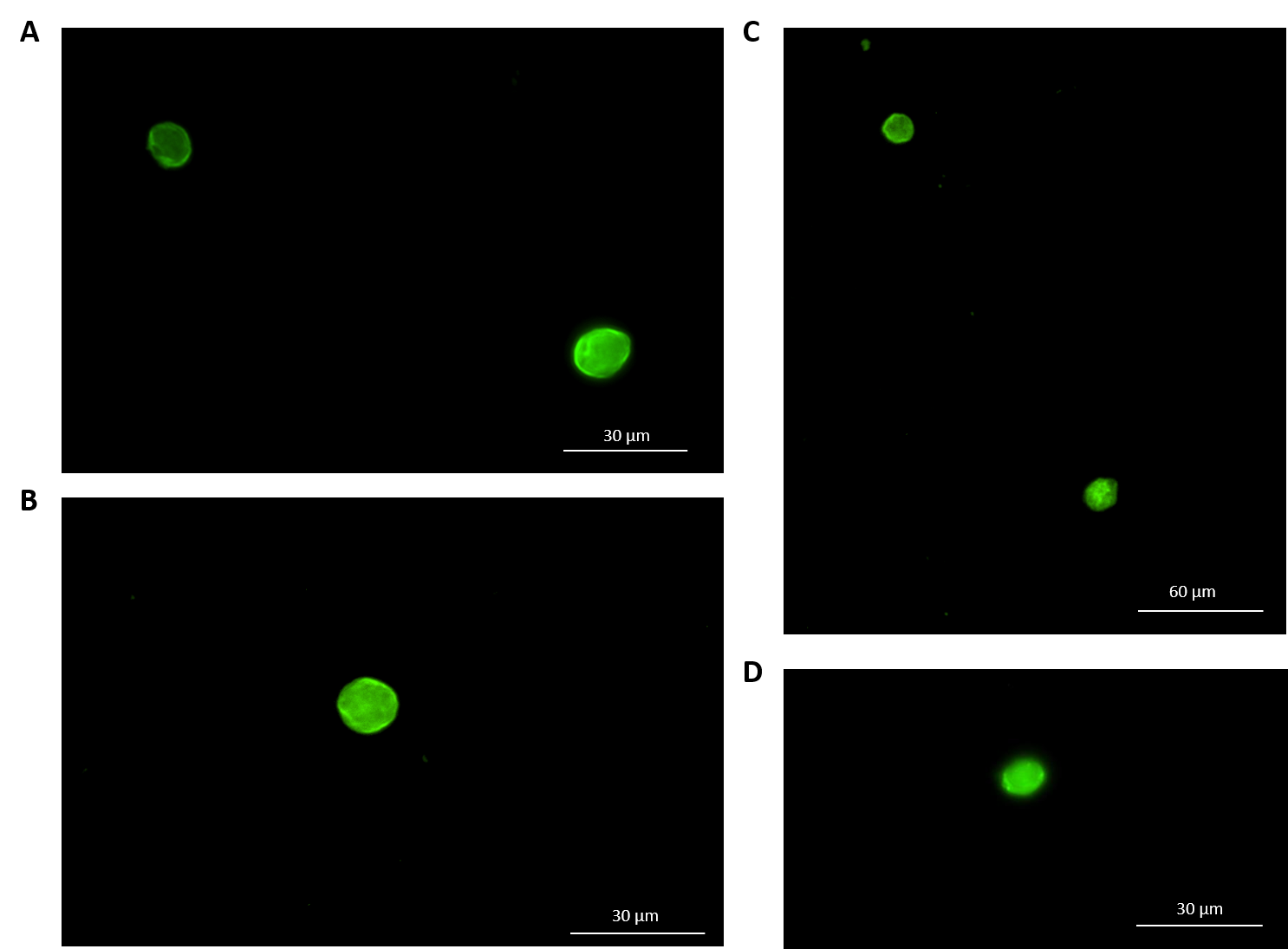

Supplement: Supplementary Figure 4 — Immunofluorescence pictures of mature cysts in the Type II isolates TgShSp1 (A), TgShSp3 (B) and TgShSp2 (C), and Type III TgShSp24 (D) at low passage, stained with FITC-conjugated Dolichos biflorus lectin. The scale bar indicates the size in µm. [file Image4.tif]

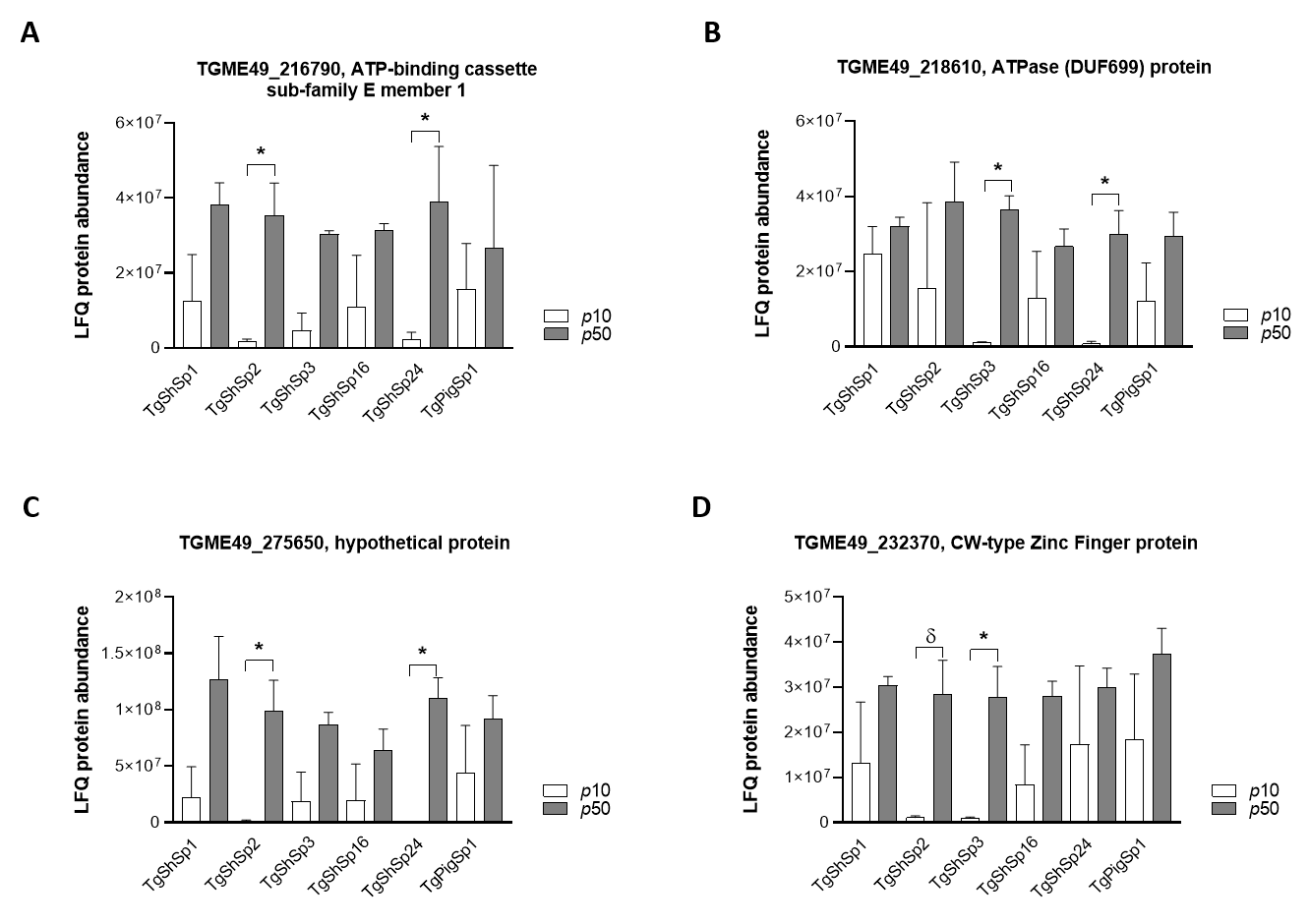

Supplement: Supplementary Figure 5 — Quantification of four differentially abundant (DA) proteins with significantly higher levels at high vs. low passages located at parasite nucleus/nucleolus in Type II and Type III canonical isolates: ATP-binding cassette sub-family E member 1 (A), ATPase (DUF699) protein (B), hypothetical protein TGME49_275650 (C) and CW-type Zinc Finger protein (D). Columns represent the mean, and bars the standard deviation of label-free quantification (LFQ) values for three biological replicates for each isolate at low (p10) or high (p50) passages (see legend, p10 = p10-p16; p50 = p50-p53). * indicates significant DA proteins by iLFQ and iTOP3 levels. δ indicates significant DA protein by only either iLFQ or iTOP3 levels (log2 fold-change < -1 or >1; p-adjusted < 0.05). Note that LFQ abundance scale in y-axis varies among charts for easier visualization. [file Image5.tif]

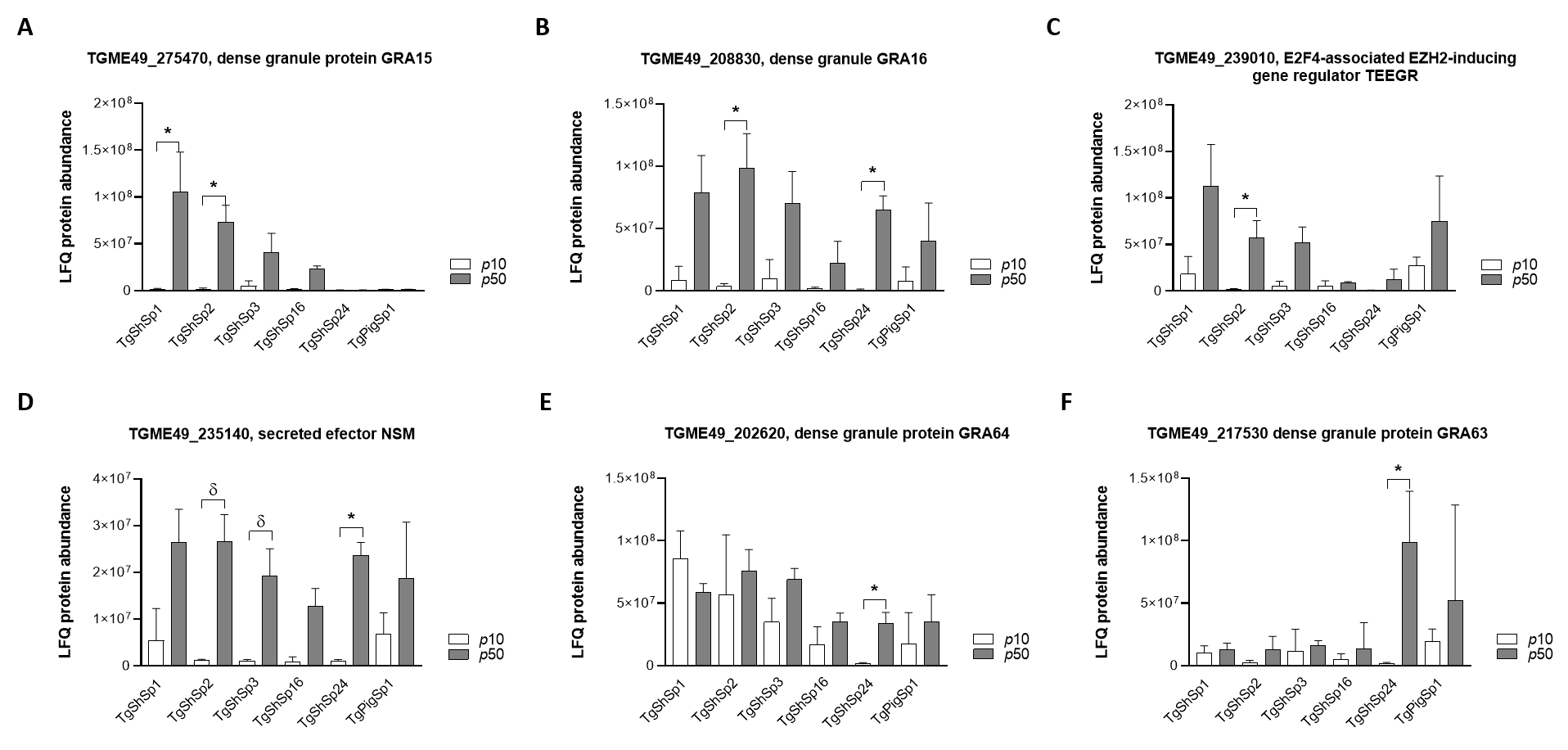

Supplement: Supplementary Figure 6 — Quantification of six differentially abundant (DA) proteins with significantly higher levels at high vs. low passages secreted from dense granules in Type II and Type III canonical isolates: GRA15 (A), GRA16 (B), E2F4-associated EZH2-inducing gene regulator TEEGR (C), secreted effector protein NSM (D), GRA64 (E) and GRA63 (F). Columns represent the mean, and bars the standard deviation of label-free quantification (LFQ) values for three biological replicates for each isolate at low (p10) or high (p50) passages (see legend, p10 = p10-p16; p50 = p50-p53). * indicates significant DA proteins by iLFQ and iTOP3 levels. δ indicates significant DA protein by only either iLFQ or iTOP3 levels (log2 fold-change < -1 or >1; p-adjusted < 0.05). Note that LFQ abundance scale in y-axis varies among charts for easier visualization. [file Image6.tif]

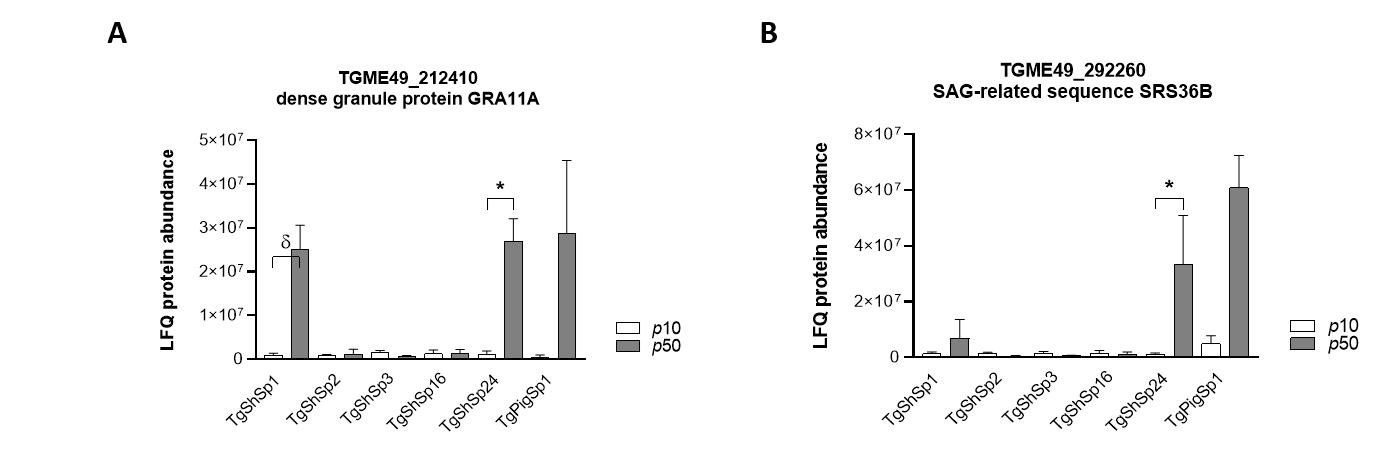

Supplement: Supplementary Figure 7 — Quantification of two differentially abundant (DA) proteins with significantly higher levels at high vs. low passages in the Type III TgSpSp24 isolate related to merozoite stage: GRA11A (A) and SRS36B (B). Columns represent the mean, and bars the standard deviation of label-free quantification (LFQ) values for three biological replicates for each isolate at low (p10) or high (p50) passages (see legend, p10 = p10-p16; p50 = p50-p53). * indicates significant DA proteins by iLFQ and iTOP3 levels. δ indicates significant DA protein by only either iLFQ or iTOP3 levels. Note that LFQ abundance scale in y-axis varies among charts for easier visualization. [file Image7.tif]
